# Sodium-glucose cotransporter 2 inhibitors in cardiology: Slow start and sweet passage

**DOI:** 10.1007/s12471-021-01618-y

**Published:** 2021-08-31

**Authors:** R. J. G. Peters

**Affiliations:** Department of Cardiology, Amsterdam University Medical Center, Academic Medical Center/University of Amsterdam, Amsterdam and VUmc/Free University, Amsterdam, The Netherlands

Sodium-glucose cotransporter 2 (SGLT2) inhibitors suppress proximal tubular reabsorption of glucose in the kidney. By this mechanism, they reduce plasma glucose concentrations and increase the excretion of glucose in urine, irrespective of the presence of diabetes mellitus. In fact, by inducing glucosuria, in a literal sense (and literally only) these drugs cause diabetes mellitus: the honeysweet passage of urine.

Multiple clinical trials in patients with heart failure, with or without type 2 diabetes, have demonstrated that SGLT2 inhibitors reduce the risk of major adverse cardiovascular events, including hospitalisation for heart failure and cardiovascular death, and that they inhibit the progression of kidney function loss. In addition, they are associated with reduced symptoms and improvements in quality of life. These benefits are seen both in patients with and without type 2 diabetes. The risks of adverse events in the natural course of disease in these cardiovascular patients are very high, and there is an obvious need for effective prevention. The risk reductions achieved with SGLT2 inhibitors in these patients are clear and consistent, and the drugs are associated with few side effects. In addition, a single tablet a day will do [1, 2].

Based on the trial evidence and recent guidelines, widespread introduction into the practice of cardiology would be expected. However, uptake is slow, and it appears that cardiologists in the Netherlands are more hesitant than colleagues in other countries. This has also been observed with the introduction of other pharmacological innovations, such as direct oral anticoagulants [3].

Several barriers to the introduction of these new agents may play a role. The addition of yet another drug to the polypharmacy in these patients likely impacts the compliance of both patients and physicians. There may be a limit to the number of treatments that both parties can oversee, comprehend or accept. Preventive treatments in general are more difficult for patients to accept than treatments that relieve symptoms. A major drawback in prevention is that its rewards are invisible in the individual patient: nothing happens if prevention is effective. In the case of SGLT2 inhibitors, however, there is also a favourable effect on symptoms, quality of life and functional status. This should facilitate the acceptance by both patients and physicians.

For clinicians, each addition to the drug schedule of a patient increases their workload, including the review of indications and contraindications, mandatory administration, consultation of other disciplines, mandatory lab work and follow-up visits. Not all cardiologists are familiar with the titration of antidiabetic medication, and drug changes may disturb a satisfactory balance. Costs may play a role; however, in the case of SGLT2 inhibitors, this is not a critical issue, at 1.50 euro a day.

In this issue of the *Netherlands Heart Journal*, Zwart et al. present a brief review of the literature and a practical approach to starting SGLT2 inhibitors in patients, for use in everyday cardiology practice [4]. The authors build on trial evidence and guidelines to create a highly practical manual for daily use. Hopefully, this will stimulate clinicians to offer SGLT2 inhibitors to patients in whom the benefits may be expected. In addition, the authors report on their initial experience in 84 patients. This provides a real-world impression of what cardiologists may expect when introducing these agents into their practice.

Based on these observations, it appears to be helpful to initiate centre-based clinical routines and pathways that include the participation of cardiologists, internists and paramedical personnel. A protocol and standardised procedures will facilitate the adoption of this new class of drugs and help to secure a sweet flow in the clinician’s office.
